# Personalizing Mobile Apps for Health Behavioral Change According to Personality: Cross-Sectional Validation of a Preference Matrix

**DOI:** 10.2196/78939

**Published:** 2026-04-22

**Authors:** Laetitia Gosetto, Gilles Falquet, Frederic Ehrler

**Affiliations:** 1 Research Institute for Statistics and Information Science, Geneva School of Economics and Management, University of Geneva, 40 Boulevard du Pont-d'Arve, Geneva, 1211, Switzerland, 41 3726288; 2 Division of Medical Information Sciences, University Hospital of Geneva, Geneva, Switzerland; 3 Information Systems Directorate, University Hospital of Geneva, Geneva, Switzerland

**Keywords:** mobile health, mHealth, behavior change technique, personalization, personality, Big Five

## Abstract

**Background:**

Mobile health (mHealth) apps are increasingly used to support healthy lifestyle behaviors through features such as health tracking and personalized reminders. Personalized messaging, tailored to users’ profiles, has been shown to improve engagement and retention in health-related contexts. Prior research has linked personality traits, based on the Big Five model, to preferences for specific app mechanisms, leading to the development of a preference matrix for personalizing mHealth apps. This matrix comprises 15 mechanisms derived from behavior change techniques and gamification elements, intended to guide developers in optimizing engagement according to user profiles.

**Objective:**

This study aimed to validate this preference matrix by examining whether the associations between mechanisms and Big Five personality traits reported in the literature align with user preferences observed in an experimental setting.

**Methods:**

A cross-sectional study was conducted using an online survey that collected demographic data, mHealth app usage, and personality traits. Participants were presented with mockups illustrating 15 mechanisms and were asked to select their preferred options. Logistic regression and ordinal logistic regression analyses were performed to examine associations between personality traits, mechanism selection, and motivation scores. All analyses were adjusted using the Bonferroni correction to account for multiple comparisons.

**Results:**

A total of 214 participants completed the survey (mean age 29.42, SD 10.41 y; n=118, 55.1% women; n=89, 41.6% men; n=5, 2% identifying as other; and n=2, 1% nonrespondents). Higher conscientiousness significantly increased the likelihood of selecting the collection mechanism (eg, collecting badges or points; odds ratio [OR] 1.87, 95% CI 1.27-2.75). For competition (eg, competing with other users), conscientiousness (OR 3.22, 95% CI 1.73-6.00) and agreeableness (OR 1.93, 95% CI 1.08-3.45) were significant predictors. Preferences for rewards (eg, virtual incentives such as points or virtual currency) were associated with conscientiousness (OR 2.36, 95% CI 1.53-3.63) and neuroticism (OR 1.97, 95% CI 1.36-2.86). Additionally, 4 mechanisms—self-monitoring, progression, challenge, and quest—were selected by more than half of the participants, independent of personality traits.

**Conclusions:**

The findings partially validate the proposed preference matrix. Conscientiousness consistently emerged as a key predictor of preference across multiple mechanisms, highlighting its central role in engagement with gamified mHealth features. While some mechanisms appear to have universal appeal, others show personality-specific preferences, underscoring the value of combining baseline mechanisms with targeted personalization strategies in mHealth app design.

## Introduction

### Background

Many studies have demonstrated the role of healthy lifestyle behaviors in increasing life expectancy. Specifically, the adoption of a healthy diet, maintenance of a healthy weight, cessation of smoking, consumption of alcohol in moderation, and regular exercise are associated with significant reductions in mortality. The researchers discovered that the adoption of each healthy lifestyle behavior contributes to an improved quality of life and enhanced longevity [1].

The number of health apps designed to assist individuals in adopting healthier behaviors has continued to grow steadily, with new apps reaching the market every year. In 2018, there were over 35,000 health apps available for download [2]. The advent of smartphone apps has created new opportunities for engaging in health-promoting behaviors. These apps offer immediate access to health-related information, medication reminders, and tools for monitoring progress, which can collectively support healthier lifestyle adoption [3].

Several scales have been developed to assess the quality of mobile health (mHealth) apps, such as the Mobile App Rating Scale [4] and the App Behavior Change Scale [5]. A key feature shared across these frameworks is the inclusion of personalization as a critical quality dimension. Personalization is widely acknowledged as essential in the design of behavioral interventions. For example, 1 study has demonstrated that messages tailored to the user’s specific characteristics are more likely to be read, recalled, and retained in memory, and are perceived as more personally relevant than untailored messages. Tailored messages also attract greater attention, stimulate more discussion, and are shared more frequently compared to nontailored messages [6].

### The Preference Matrix

Personalization of mobile apps can be done according to the user’s profile. In previous studies [7], we identified preference relations between personality traits and specific mechanisms (components that can be integrated into an mHealth app) based on a scoping review of the literature. These findings led to the development of a preference matrix indicating the types of mechanisms preferred according to a user profile. The preference matrix can be used as a framework for the personalization of mobile apps with the aim of encouraging behavioral change. When designing a mobile app intended to support health-related behavior change, designers can draw on this preference matrix to identify the mechanisms most likely to be effective for their target users. For instance, if an individual is classified as extraverted according to the Big Five personality traits, mechanisms that facilitate social comparison or collaboration between users may be particularly relevant [8].

The preference matrix includes 15 mechanisms associated with the Big Five personality traits, which are presented in detail in the table found in Multimedia Appendix 1. For personality profiling, we used one of the most widely applied classification systems, namely the Big Five personality model [8-16].

### Personality Measure: The Big Five

One of the most widely used instruments for the assessment of personality is the Big Five model. The Big Five model describes an individual’s personality based on 5 distinct traits, as summarized in Table 1.

This model has the advantage of being widely adopted in academic research. Between 1990 and 1994, the number of publications on this topic was approximately 400, while between 2005 and 2009, this figure rose to approximately 1600 [17].

The other advantage of this model is its cross-cultural universality. The Big Five model has been shown to be universal across human populations [18]. Indeed, the authors translated their personality questionnaire, the Revised NEO Personality Inventory (NEO-PI-R), into 6 languages belonging to the same language family as English (German, Portuguese, Hebrew, Chinese, Korean, and Japanese), and the Big Five factors consistently emerged across these versions.

In a subsequent study, researchers examined whether the Big Five personality dimensions, as measured by the NEO-PI-R, were universal across 50 cultures spanning American, European, Arabic, Asian, and African regions [19]. The NEO-PI-R was translated into the local language of each participating country. The participants were primarily students from the country in which the test was administered. Factor analysis demonstrated that the American structure of the NEO-PI-R self-report questionnaire (Form S) could be replicated in all cultures, with 94.4% of factors replicated. Moreover, the general factor structure remained recognizable across all cultural contexts included in the study.

**Table 1.** Definition of the Big Five factors of personality.

Traits	The 5 traits represent the tendency to... [20]
Neuroticism	Worried, nervous, emotional, anxious, maladjusted, and hypochondriac
Extraversion	Sociable, active, talkative, open to others, optimistic, fun-loving, and affectionate
Openness	Curious, eclectic, creative, original, imaginative, and nonconformist
Agreeableness	Compassionate, easy-going, trusting, helpful, indulgent, credulous, and honest
Conscientiousness	Organized, reliable, hard-working, disciplined, punctual, meticulous, careful, ambitious, and persevering

### Selection of the 15 Mechanisms

Based on the hypothesis that individuals with different profiles exhibit distinct preferences regarding components of mHealth apps designed to facilitate behavior change, a review of the literature identified 15 mechanisms associated with user profiles [21]. In our context, the term “mechanism” is used to denote the entirety of components that can be incorporated within an mHealth app with the objective of promoting behavioral change. We classified the mechanisms into 2 categories: the mechanisms linked to behavior change techniques and the mechanisms linked to game elements. For further details and definition of mechanisms, see Table 2.

**Table 2.** List of mechanisms with their definitions.

Mechanism	Definition
Behavior changes techniques	
Prompt and cues	Usually, a message delivered to the user to prompt or recall a behavior at a specified time, with the app or user defining when the message should be sent [22]
Demonstration of the behavior	Enables users to observe the cause-and-effect linkage of their behavior, such as seeing a simulation of their bodies after a diet [23]
Self-monitoring	Users can track their behaviors, providing information on both past and current activities [23]
Punishment	Virtually penalizes the user for not performing the desired behavior or reaching their goal [10]
Social comparison	An individual’s perceptions of the prevailing beliefs and behaviors within a social group
Social support	Enables communication between users, such as through chat or sharing activities with other users [24]
Game elements	
Progression	Users can track their progression with steps through the system’s purpose over time, visualized with mechanisms like stars or flags along a path [23]
Competition	Users can compete to accomplish the desired behavior [23]
Cooperation	Users collaborate to achieve a shared objective [23]
Collection	Allows users to gather virtual objects
Rewards	Virtual rewards offered to users for engaging in the target behavior [23]
Quest	Users can enter or define the objectives targeted for the activity they will perform [22]
Challenge	Presents various situations that require effort from the user to be completed [24] (eg, accomplishing 3 hours of physical activity per week)
Avatar	Allows users to share their data in the system without revealing their name [24]
App mechanism	
Customization	In contrast to personalization—which involves adjusting automatically the system to the user—customization refers to the user’s ability to modify the content or functionalities of the mobile app according to their own preferences [23]. This approach enables users to actively tailor the system based on users’ choices.

### Preferences Relations Between Big Five Traits and Mechanisms

The preference relations between the mechanisms and the Big Five traits identified in the literature enable us to construct a preference matrix summarized in Multimedia Appendix 1. Each cell of the matrix indicates the number of studies in the review reporting a preference relation between a given user profile and a specific mechanism [21]. Most of the preference relations were positive (53/75, 71%), indicating that a particular personality profile tends to prefer a given mechanism (represented by a plus sign in Multimedia Appendix 1). Negative relations, representing an aversion toward a mechanism (represented by a minus sign in Multimedia Appendix 1), accounted for 12% (9/75) of all potential relations. It is important to note that the relation matrix remains incomplete, as only 73% (55/75) of all potential relations were identified in the literature review.

This study aims to corroborate or enrich this preference matrix by experimentally identifying new preference or nonpreference relations between mechanisms and personality traits.

## Methods

### Ethical Considerations

This study was approved by the University of Geneva Research Ethics Committee (CUREG_2021-04-38). This section is based on our previously published protocol study [25]. Participants were required to complete a consent form that detailed the purpose of the study, the procedure to be followed, and their right to withdraw from the study at any time. Confirmation that they had read, understood, and agreed to the terms of the consent form was mandatory before accessing the questionnaire and was required for the investigators to use the submitted data for research purposes. Participation was voluntary, responses were collected anonymously, and only deidentified data were used for research purposes in accordance with applicable data protection regulations. Participants did not receive any financial compensation for their participation.

### Study Design

We conducted a cross-sectional study to address our research objectives. Participants completed an online questionnaire following the CHERRIES (Checklist for Reporting Results of Internet E-Surveys) guidelines (Checklist 1) [26].

### Outcomes

The primary outcome was the identification of preferred mechanisms according to the user profile.

### Study Population

The study targeted French-speaking adults aged 18 years and older. Recruitment was conducted both within the University of Geneva community and through social media platforms (eg, Facebook and Twitter, subsequently rebranded X), enabling participation from a broader population beyond university affiliates. The survey was open to anyone meeting the language and age criteria.

### Sample Size Calculation

We calculated the required sample size using a multiple regression power analysis in R (R Foundation for Statistical Computing). The parameters used were the number of predictors (u=3), effect size (*f*²=0.07), significance level (*α*=.05), power of 0.9, and estimated variance of 202.403. These estimates were based on the hypothesis that individuals with higher levels of agreeableness, as measured by the Big Five personality traits, show a preference for social networks [11,24].

To approximate the variance, we used data from a previous study [27] that assessed Big Five personality traits in relation to user preferences for social network posters. Specifically, we relied on the variance in Big Five trait ratings for altruistic participants (n=46) based on their average scores for a blood donation poster, which was evaluated on a 0-to-100 scale. Based on these parameters, we determined that a minimum sample size of 206 participants was required.

### Procedure

Participants completed an online questionnaire developed by the investigators using Qualtrics software (Qualtrics; Multimedia Appendix 2). First, participants were required to complete a consent form that detailed the purpose of the study, the procedure to be followed, and their right to withdraw from the study at any time. Participants were then asked to provide demographic information. To proceed to the remainder of the survey, they were required to confirm that they were aged 18 years or older. Eligible participants subsequently completed two main components of the questionnaire: (1) a standardized personality assessment measuring their Big Five profile, and (2) a task in which they reviewed 15 mechanisms, selected their 5 preferred ones, and rated the extent to which each selected mechanism would motivate them to engage in physical activity on a scale from 0 to 100.

### Measures and Measurement

#### Demographic Questions

The participants were asked to provide information regarding their gender, age, occupation, and level of education.

#### Profile Assessment

To assess the personality of the participants, we used the French version of the Big Five Inventory-10 (BFI-10-Fr) scale, translated and validated by Courtois [26]. The internal reliability of the Big Five Inventory-10 (BFI-10) is reported to be relatively low, with Cronbach α coefficients ranging from 0.37 to 0.83. However, this limitation is expected, as Cronbach α is not well-suited for very short scales, particularly those with only 2 items per dimension [26]. The instrument consists of 10 items, with 2 items pertaining to each of the 5 Big Five dimensions. Participants were requested to indicate on a 5-point Likert scale whether they approve or disapprove of the statements pertaining to themselves. For instance, respondents indicated whether they perceived themselves as reserved or as someone who is easily anxious. Scores for each personality dimension were computed by summing the two relevant items, with reverse scoring applied where appropriate.

This scale was selected because its factorial structure mirrors that of the full BFI-10-Fr, providing a valid measure of personality while minimizing participant burden [26]. Given the number of instruments included in the overall protocol, using a short, validated scale was deemed necessary to reduce fatigue and prevent attrition associated with excessively long questionnaires.

#### Choice of Mechanisms

Each of the 15 gamification mechanisms was represented by a mockup accompanied by a short textual description (Multimedia Appendix 2). To minimize potential bias related to visual design preferences, all mockups were created using a deliberately simple and neutral layout. The interface used only black-and-white tones and standardized icons to ensure clarity and comparability across mechanisms (Multimedia Appendix 2). The 15 mockups were displayed in a randomized order to control for possible primacy or recency effects. Participants were instructed to carefully review each screen and its corresponding description before making their choices. After viewing all 15 mechanisms, they were asked to select the five they personally found most motivating.

#### Explanation of Choice

For each of the 5 mechanisms selected in the previous step, participants were asked to indicate the degree to which that specific mechanism would motivate them to adopt healthier behaviors, using a continuous scale ranging from 0 (not at all motivating) to 100 (extremely motivating). After rating each of their 5 chosen mechanisms, participants were also invited to provide an open-ended written explanation describing the reasons behind their selections. All mechanisms that were not included among the participants’ 5 preferred choices were automatically assigned a score of zero.

### Analysis

#### Preference Matrix Validation

To compare the experimental results with the preference patterns reported in the literature, three matrices were generated.

The first matrix (M1) summarizes the preference relationships identified in the scoping review previously conducted [21]. Each cell represents the strength of evidence supporting a link between a Big Five personality trait and a gamification mechanism. The numerical value indicates the number of studies reporting a positive association (eg, 1=one study, 2=two studies, 3=three studies, and so on). A value of –1 denotes an aversion relationship, 0 indicates that both preference and aversion were observed, and null corresponds to an absence of a reported relationship.

The second matrix (M2) was derived from the empirical data collected in this study. Each cell represents a discriminative value computed for the combination of a Big Five trait (column) and a gamification mechanism (row). This value reflects the difference between the average personality scores of participants who selected a given mechanism and those who did not, as calculated using the following formula:$C_{m,b}=round(\frac{\sum_{p=1}^{n}1_{p,m}\cdot S_{p,b}}{\sum_{p=1}^{n}1_{p,m}}-\frac{\sum_{p=1}^{n}0_{p,m}\cdot S_{p,b}}{\sum_{p=1}^{n}0_{p,m}})$ (1)

where 1_p,m_=1 if participant p selected mechanism m, 0_p,m_=1 if participant p did not select mechanism m, S_p,β_ is the score of participant p on the Big Five dimension _β_, and n is the total number of participants.

To facilitate interpretation, discriminative values were categorized based on their magnitude: 0‐1 (minimal selection of the mechanism by that trait), 2 (moderate selection), 3 (high selection), and 4+ (very high selection).

The third matrix (M3) represents the difference between M1 and M2, allowing direct visual and quantitative comparison between literature-based expectations and experimental results. Positive values indicate stronger associations in the current data than previously reported, whereas negative values indicate weaker or opposing trends. Cells with “null” values in M1 were excluded from the difference calculations.

#### Statistical Analysis

A logistic regression analysis was conducted to examine the relationship between the mechanisms and the Big Five personality dimensions. The purpose of this analysis was to determine whether participants’ scores on the BFI-10-Fr significantly predicted the likelihood of selecting a given mechanism. Accordingly, logistic regression analysis was conducted for each mechanism to ascertain whether the BFI-10-Fr scores were predictive of the mechanism selection.

In addition, an ordinal logistic regression analysis was performed using the motivation scores assigned to the selected mechanisms as dependent variables and the BFI-10-Fr scores as predictors. A distinct model was computed for each mechanism to evaluate whether personality scores predicted the level of motivation attributed to that mechanism.

To reduce the risk of type I errors, the Bonferroni correction was applied across all regression analyses. This adjustment was implemented to account for the multiple comparisons conducted across mechanisms and personality dimensions, thereby minimizing the likelihood of false positive results.

## Results

### Demographics Data

A total of 214 participants completed the questionnaire, including 118 (55.1%) women, 89 (41.6%) men, 5 (2%) individuals identified as “other,” and 2 (1%) participants who did not report their gender. The mean age of the sample was 29.42 (SD 10.41) years. Additional demographic characteristics are presented in Table 3.

**Table 3.** Demographics data. Participants were recruited both within the University of Geneva community and through social media platforms, allowing for the inclusion of individuals beyond the academic environment (N=214).

Characteristics	Values	
Age (y), mean (SD)	29.42 (10.41)	
Gender (N=214), n (%)		
Women	89 (41.6)	
Men	118 (55.1)	
Others	7 (3.3)	
Education level (n=211^a^), n (%)		
Mandatory education	53 (24.8)	
Bachelor’s degree	63 (29.4)	
Master’s degree	80 (37.4)	
Doctorate	15 (7)	
Smartphone use (N=214), n (%)		
Not comfortable	10 (4.7)	
Comfortable	204 (95.3)	
Already used mHealth^b^, n (%)	135 (63.1)	

a Education level: 3 missing responses (n=211).

b mHealth: mobile health.

### Characteristics of the Participants on the Big Five

**Figure 1.**
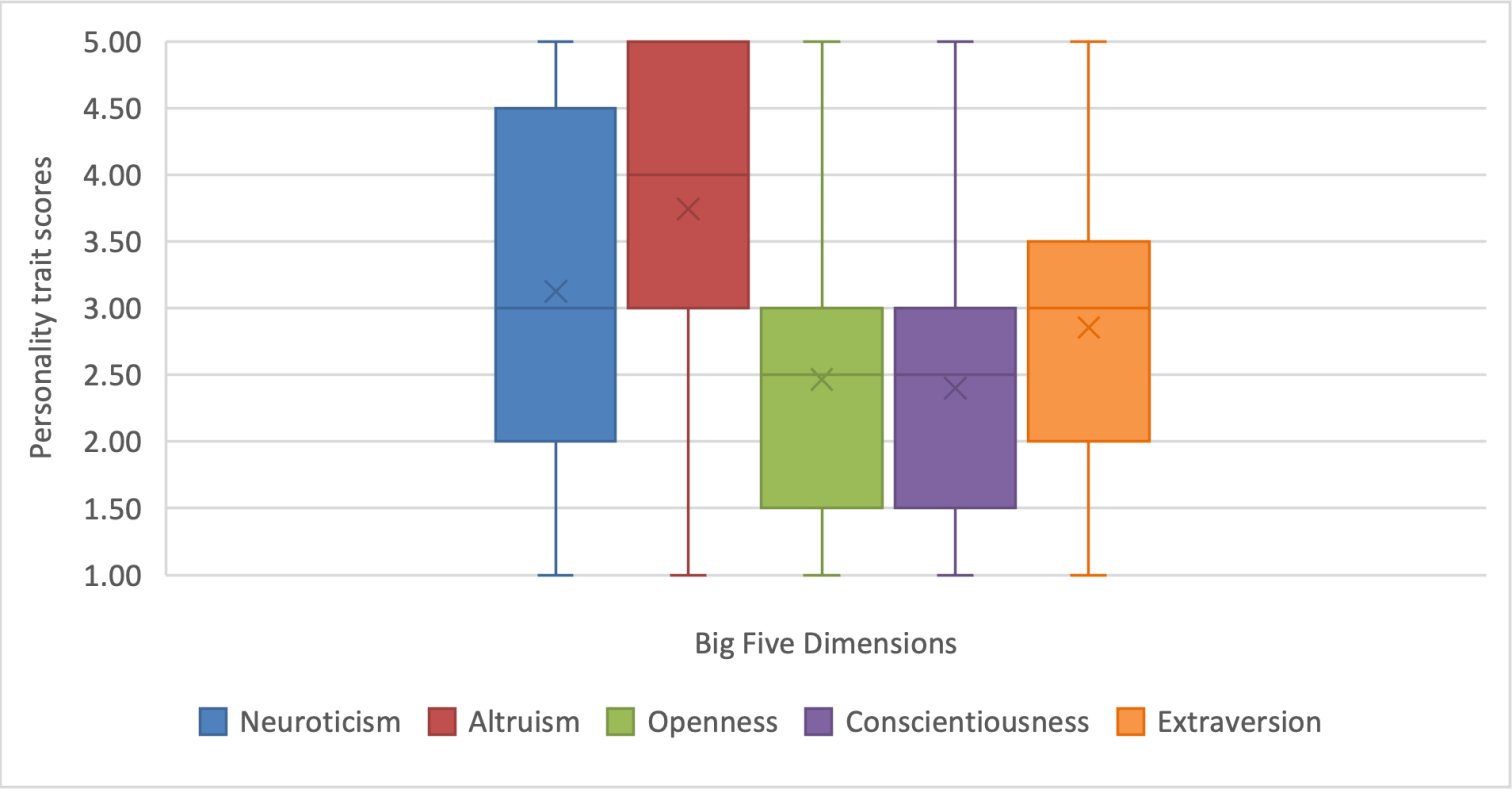
Boxplot of the representativeness of the participants on the Big Five (N=214).

Personality trait scores were measured on a 1 to 5 scale. The distribution of the sample across the Big Five dimensions is presented in Figure 1, where the line depicts the median and the cross indicates the mean.

Overall, participants in this study tended to score higher on agreeableness (mean 3.71, SD 1.31), followed by neuroticism (mean 3.12, SD 1.23) and extraversion (mean 2.86, SD 1.17).

### Characteristics of Participants’ Selections for Each Mechanism

A total of 4 mechanisms were selected by more than half of the participants. These mechanisms were self-monitoring, progression, challenge, and quest (Table 4).

**Table 4.** Selection frequency of each mechanism and mean Big Five trait scores among participants (N=214).

Mechanisms	People who selected the mechanism, n (%)	Neuroticism, mean (SD)	Agreeableness, mean (SD)	Openness, mean (SD)	Conscientiousness, mean (SD)	Extraversion, mean (SD)
Self-monitoring	149 (69.6)	3.1 (1.2)	3.8 (1.3)	2.5 (1.0)	2.3 (1.0)	2.9 (1.1)
Progression	127 (59.3)	3.1 (1.2)	3.7 (1.3)	2.6 (1.0)	2.3 (0.9)	2.9 (1.2)
Challenge	112 (52.3)	3.2 (1.6)	3.7 (1.4)	2.4 (1.0)	2.4 (1.0)	2.9 (1.2)
Quests	111(51.9)	3.0 (1.2)	3.8 (1.3)	2.6 (1.0)	2.3 (0.9)	2.9 (1.2)
Cooperation	70 (32.7)	3.1 (1.2)	3.8 (1.3)	2.3 (1.0)	2.3 (0.9)	3.0 (1.3)
Demonstration of the behavior	61 (28.5)	3.2 (1.2)	3.6 (1.3)	2.3 (0.9)	2.3 (1.0)	2.7 (1.2)
Prompt and cues	53 (24.8)	3.3 (1.3)	3.6 (1.3)	2.6 (1.1)	2.4 (0.9)	2.8 (1.2)
Rewards	47 (2%)	3.4 (1.4)	3.6 (1.3)	2.5 (1.0)	2.8 (1.0)	2.8 (1.3)
Social comparison	44 (20.6)	3.2 (1.2)	3.7 (1.3)	2.7 (1.0)	2.6 (0.9)	2.8 (0.9)
Collectible	41 (19.2)	2.9 (1.3)	3.5 (1.6)	2.5 (1.0)	2.7 (1.0)	3.0 (1.2)
Avatar	29 (13.6)	3.2 (1.3)	3.8 (1.5)	2.6 (0.9)	2.5 (0.8)	3 (1.3)
Competition	29 (13.6)	2.8 (1.3)	3.9 (1.4)	2.4 (0.9)	2.8 (1.1)	2.7 (1.2)
Social support	20 (9.3)	2.9 (1.2)	4.0 (1.0)	2.5 (1.0)	2.4 (1.1)	2.9 (1.1)
Punition	8 (3.7)	3.3 (1.5)	3.5 (1.3)	2.2 (0.7)	2.3 (1.0)	3.1 (1.2)

### Comparison Between This Study’s Results and the Preference Matrix

**Figure 2.**
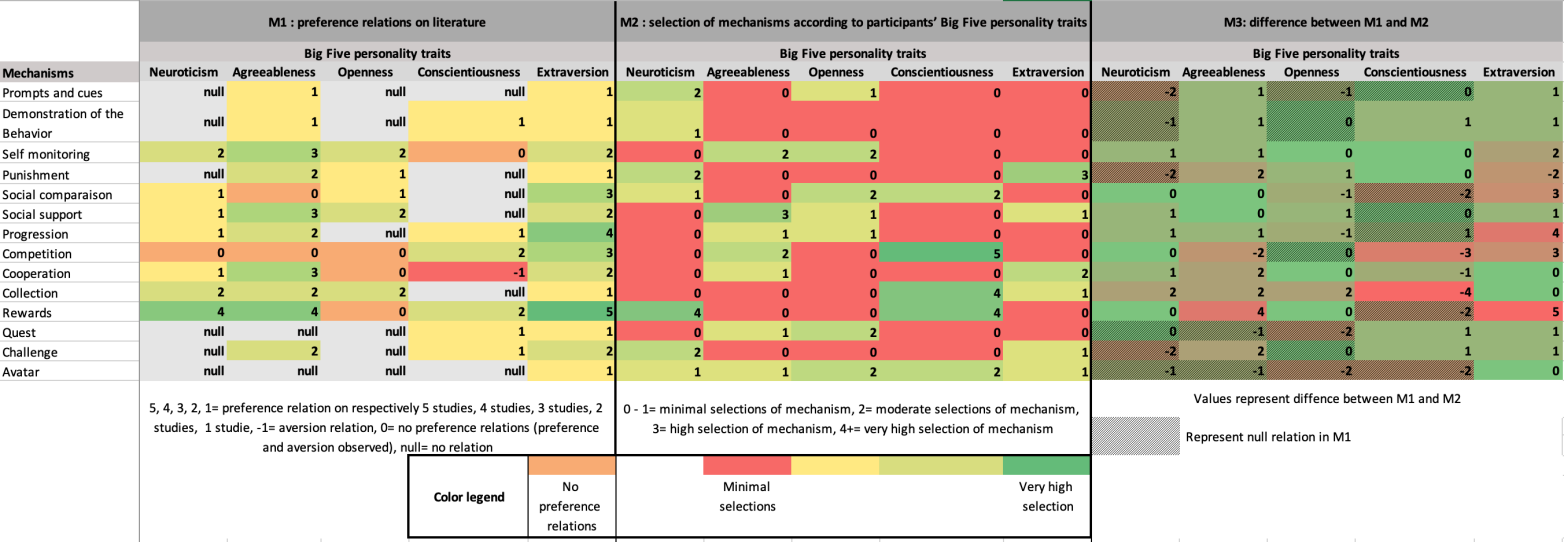
Matrices for the representation of preference relations in literature (M1), the selection mechanisms used by participants based on their Big Five traits scores (M2), and the difference between M1 and M2 (M3).

The initial matrix (M1) summarizes the preference relations identified in the literature between personality traits and mechanisms. A positive relation indicates that individuals with a given Big Five profile tend to prefer a particular mechanism, whereas a negative relation (aversion) reflects a lower interest or dislike for that mechanism among individuals with that profile. Mixed relations correspond to cases where both preference and aversion were reported across studies, while missing relations denote the absence of evidence. Overall, M1 presents 60% (42/70) positive preference relations, 1% (1/70) aversion relations, 10% (7/70) mixed relations, and 29% (20/70) missing relations (see M1 in Figure 2).

The second matrix (M2) represents the empirical data collected in this study. For each selection mechanism (eg, collection, challenge, or reward), a discriminative score was calculated. This score indicates how strongly a given personality trait differentiates between participants who selected a mechanism and those who did not. M2 shows that 5% (4/70) of mechanisms had a very high discriminative score (>4), 3% (2/70) high (3), 17% (12/70) moderate (2), and 74% (52/70) low (0‐1; see M2 in Figure 2).

The second matrix (M2) represents the empirical data collected in this study. For each selection mechanism—defined as one of the gamified motivational strategies participants could choose (eg, collection, challenge, or reward)—a discriminative score was calculated. This score indicates how strongly a given personality trait differentiates between participants who selected a mechanism and those who did not. M2 shows that 5% (4/70) of mechanisms had a very high discriminative score (>4), 3% (2/70) high (3), 17% (12/70) moderate (2), and 74% (52/70) low (0‐1; see M2 in Figure 2).

### Preferred Mechanisms and Big Five: Statistical Analysis

A separate logistic regression analysis was performed for each mechanism to determine whether the personality scores predicted the likelihood of selecting that mechanism. In addition, an ordinal logistic regression was conducted using the motivation ratings assigned to the selected mechanisms as the dependent variables and the BFI-10-Fr scores as the predictors. Across analyses, 3 mechanisms yielded statistically significant models.

#### Collection Mechanism

For the collection mechanism, defined as a feature that allows users to gather virtual objects, the regression model was statistically significant *(χ*²_5_=13.07; n=207; *P*<.05), indicating that the model successfully distinguished between participants who selected the collection mechanism and those who did not. The model explained between 6% (Cox and Snell *R*^2^) and 10% (Nagelkerke *R*^2^) of the variance in mechanism selection and correctly classified 83.1% of cases. As shown in Table 5, only 1 predictor made a unique statistically significant contribution: conscientiousness with an odds ratio (OR) of 1.87 (95% CI 1.27-2.75). This indicates that participants scoring higher on conscientiousness were over 1.87 times more likely to select the collection mechanism compared with those scoring lower on this trait, after controlling for all other predictors in the model.

**Table 5.** Logistic regression predicting the likelihood of selecting the collection mechanism.

Personality trait	B	SE	Wald chi-square (*df*)	*P* value	OR^a^ (95% CI)
Openness	–0.019	0.193	0.010 (1)	.92	0.981 (0.672-1.432)
Conscientiousness	0.625	0.198	9.987 (1)	.002	1.868 (1.268-2.752)
Extraversion	0.035	0.168	0.043 (1)	.84	1.035 (0.745-1.439)
Agreeableness	–0.165	0.146	1.279 (1)	.26	0.848 (0.637-1.129)
Neuroticism	–0.191	0.163	1.376 (1)	.24	0.826 (0.600-1.137)
Constant	–2.089	1.093	3.654 (1)	.06	0.124 (0.015-1.054)

a OR: odds ratio.

#### Competition Mechanism

The logistic regression for the competition mechanism, a feature emphasizing performance comparison or rivalry between users, was statistically significant (*χ*²_5_=31.49; n=200; *P*<.01). This result indicates that the model successfully distinguished between participants who selected this mechanism and those who did not, based on their Big Five personality scores. The regression explained between 15% (Cox and Snell *R*^2^) and 33% (Nagelkerke *R*^2^) of the variance in the selection of the competition mechanism and correctly classified 91.5% of cases. As shown in Table 6, 4 personality dimensions made a unique statistically significant contribution to the model: neuroticism, conscientiousness, extraversion, and agreeableness. Conscientiousness emerged as the strongest positive predictor (OR 3.22, 95% CI 1.73-6.00), followed by agreeableness (OR 1.93, 95% CI 1.08-3.45). In contrast, neuroticism (OR 0.43, 95% CI 0.25-0.74) and extraversion (OR 0.52, 95% CI 0.30-0.89) were negatively associated with selecting the competition mechanism, indicating that individuals with higher levels of these traits were substantially less likely to choose this feature.

**Table 6.** Logistic regression predicting the likelihood of selecting the competition mechanism.

Personality trait	B	SE	Wald chi-square (*df*)	*P* value	OR^a^ (95% CI)
Openness	–0.195	0.286	0.466 (1)	.50	0.823 (0.469-1.441)
Conscientiousness	1.169	0.318	13.532 (1)	<.001	3.220 (1.727-6.003)
Extraversion	–0.660	0.280	5.574 (1)	.02	0.517 (0.299-0.894)
Agreeableness	0.660	0.296	4.969 (1)	.03	1.934 (1.083-3.454)
Neuroticism	–0.854	0.279	9.364 (1)	.002	0.426 (0.246-0.736)
Constant	–3.680	1.798	4.187 (1)	.04	0.025 (0.001-0.856)

a OR: odds ratio.

An ordinal logistic regression was conducted for each mechanism to determine whether participants’ Big Five personality scores predicted their motivation ratings for the competition mechanism. The model for the competition mechanism was statistically significant (*χ*²_5_=11.16; N=214; *P*=.05), indicating that the predictors reliably differentiated participants’ motivation levels for engaging in mHealth apps through competition-based features. The model explained between 5% (Cox and Snell *R*^2^) and 8% (Nagelkerke *R*^2^) of the variance in the motivation scores. As shown in Table 7, 2 personality traits made unique and statistically significant contributions to the model: conscientiousness and neuroticism. Higher conscientiousness was associated with greater motivation to use competition-based features (OR 1.63, 95% CI 0.08-0.89), whereas higher neuroticism was associated with lower motivation (OR 0.71, 95% CI 0.70-0.01).

**Table 7.** Logistic regression predicting the likelihood of the score of the competition mechanism.

Personality trait	B	SE	Wald chi-square (*df*)	*P* value	OR^a^ (95% CI)
Openness	–0.072	0.207	0.121 (1)	.73	0.931 (–0.478 to 0.553)
Conscientiousness	0.487	0.206	5.586 (1)	.02	1.628 (0.083 to 0.892)
Extraversion	–0.272	0.191	2.028 (1)	.15	0.762 (–0.647 to 0.102)
Agreeableness	0.206	0.177	1.357 (1)	.24	1.229 (–0.141 to 0.553)
Neuroticism	–0.345	0.179	3.705 (1)	.05	0.708 (–0.696 to 0.006)

a OR: odds ratio.

#### Reward Mechanism

The logistic regression for the reward mechanism, a feature offering virtual incentives for engaging in the target behavior, was statistically significant (*χ*²_5_=35.29; n=204; *P*<.001). This indicates that the model successfully distinguished participants who selected this mechanism from those who did not, based on their Big Five personality traits. The model explained between 16% (Cox and Snell *R*^2^) and 27% (Nagelkerke *R*^2^) of the variance and correctly classified 81.4% of cases. As shown in Table 8, 2 personality traits made unique statistically significant contributions to the model: conscientiousness and neuroticism. Participants scoring higher on conscientiousness were 2.36 times more likely to select the reward mechanism, while those scoring higher on neuroticism were 1.97 times more likely to choose it, controlling for all other predictors.

**Table 8.** Logistic regression predicting likelihood of selecting the reward mechanism.

Personality trait	B	SE	Wald chi-square (*df*)	*P* value	OR^a^ (95% CI)
Openness	0.060	0.194	0.095 (1)	.76	1.061 (0.726-1.551)
Conscientiousness	0.858	0.220	15.262 (1)	<.001	2.360 (1.534-3.630)
Extraversion	–0.220	0.187	1.392 (1)	.24	0.802 (0.557-1.157)
Agreeableness	0.068	0.161	0.177 (1)	.67	1.070 (0.781-1.466)
Neuroticism	0.679	0.190	12.696 (1)	<.001	1.971 (1.357-2.864)
Constant	–5.919	1.327	19.897 (1)	<.001	0.003 (0.0002-0.036)

a OR: odds ratio.

The logistic ordinal regression was statistically significant (*χ*²_5_=14.36; N=214; *P*=.01), indicating that the model successfully differentiated participants according to their motivation scores for engaging with this mechanism. The model explained between 6% (Cox and Snell *R*^2^) and 9% (Nagelkerke *R*^2^) of the variance. As shown in Table 9, 2 personality traits made a unique statistically significant contribution to the model: neuroticism and conscientiousness. Conscientiousness was the strongest predictor (OR 1.66, 95% CI 0.16-0.85), followed by neuroticism (OR 1.38, 95% CI 0.03-0.61), indicating that higher levels of these traits were associated with greater self-reported motivation to use the rewards mechanism.

**Table 9.** Logistic regression predicting the likelihood of the score of the reward mechanism.

Personality trait	B	SE	Wald chi-square (*df*)	*P* value	OR^a^ (95% CI)
Openness	0.025	0.170	0.023 (1)	.88	1.026 (–0.307 to 0.358)
Conscientiousness	0.504	0.176	8.199 (1)	.004	1.66 (0.159 to 0.849)
Extraversion	–0.031	0.156	0.040 (1)	.84	0.969 (–0.336 to 0.274)
Agreeableness	–0.083	0.132	0.389 (1)	.53	0.921 (–0.342 to 0.177)
Neuroticism	0.320	0.149	4.606 (1)	.03	1.378 (0.028 to 0.61)

a OR: odds ratio.

## Discussion

### Principal Findings

The objective of this study was to validate the preference matrix derived from a previous literature review by examining whether similar relations could be observed empirically in our sample.

Four mechanisms were selected by more than half of the participants: self-monitoring (n=149), progression (n=127), challenge (n=112), and quest (n=111). No significant associations were found between these mechanisms and the Big Five personality dimensions. However, when comparing these findings with the validated preference matrix (M1), a more nuanced interpretation emerges. According to the matrix (M1), self-monitoring is generally appreciated across all Big Five profiles, with the exception of one study reporting lower preference among highly conscientious individuals. The progression mechanism also appears to be universally preferred, reinforcing its broad motivational relevance. By contrast, the preference matrix (M1) suggests that the quest mechanism is predominantly preferred by conscientious and extraverted individuals, whereas the challenge mechanism tends to be favored by users scoring high in agreeableness, conscientiousness, and extraversion. Taken together, these results indicate that certain gamification mechanisms, particularly self-monitoring and progression, possess strong, cross-profile motivational appeal. These mechanisms should, therefore, be considered foundational elements in the design of mHealth apps, providing a core layer of user engagement upon which more personalized, profile-specific interventions may be developed.

### Comparison Between M1 and M2

The comparison between matrices M1 and M2 highlights several developments in the identification of potential relationships between mechanisms and Big Five personality traits. In M2, new associations emerge that were not identified in M1, particularly between neuroticism and mechanisms such as prompts and cues, demonstration of behavior, punishment, quest, challenge, and avatar. Similarly, openness shows newly emerging association with prompts and cues, progression, quest, and challenge, while conscientiousness displays novel relations with social comparison and avatar, as well as a particularly strong association with the collection mechanism in M2. These findings reflect a refined and evolving understanding of how specific personality traits may align with distinct mechanisms when examined in an experimental context.

In addition, whereas M1 did not show a clear consensus regarding the relationships involving agreeableness and openness, M2 reveals emerging associations, notably between agreeableness and competition, and between openness and rewards. Conversely, several relationships previously identified in M1 do not reappear in M2. For example, extraversion was associated in M1 with a wide range of mechanisms, including prompts and cues, demonstration of behavior, self-monitoring, punishment, social comparison, social support, progression, competition, cooperation, collection, rewards, and quest, but these associations were not observed in M2. A similar reduction in associations is observed for agreeableness, which in M1 was linked to prompts and cues, demonstration of behavior, punishment, collection, challenge, and especially rewards. Despite these discrepancies, statistically significant results in this study were identified for only 3 mechanisms: collection, competition, and rewards. Of the 5 significant relationships observed, 3 were already present in the preference matrix M1 (conscientiousness-competition, conscientiousness-rewards, and neuroticism-rewards). Two relationships, those involving neuroticism-competition and agreeableness-competition, had shown inconsistent evidence in M1, with both preference and nonpreference relations reported in the literature.

This study contributes to refining the preference matrix in two ways. First, it enriches the matrix by identifying a new significant preference relation between conscientiousness and the collection mechanism. Second, in cases where the literature reports incongruent findings, our results help clarify these relationships by supporting a nonpreference relation for neuroticism and agreeableness with competition.

**Figure 3.**
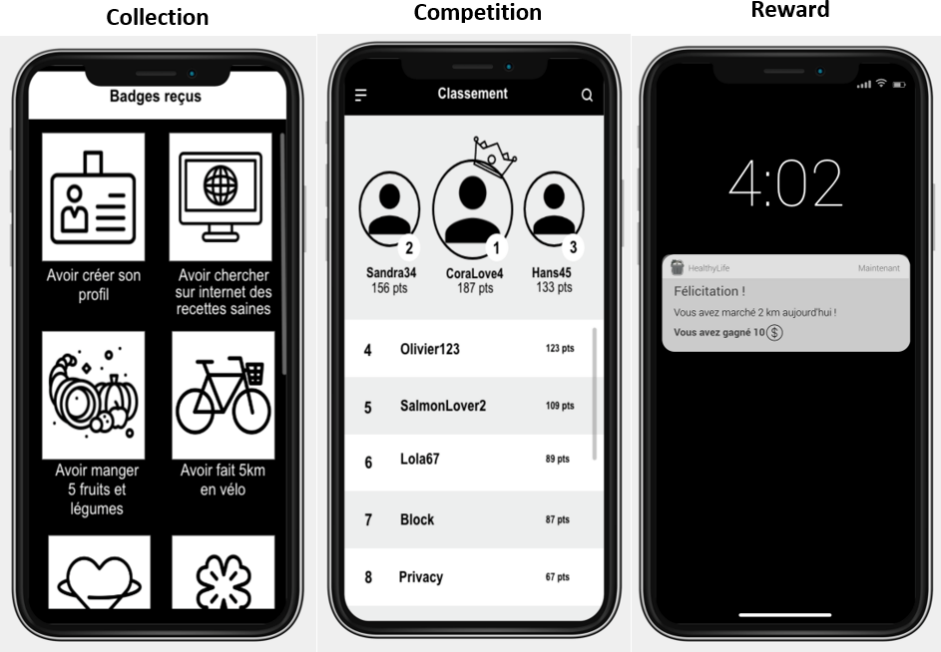
Preferred mechanisms according to personality profiles.

In the following sections, we examine in greater detail the 3 mechanisms for which significant regressions were obtained. Figure 3 presents the mockups used in the online survey to illustrate the 3 mechanisms preferred by participants according to their personality traits. These visual representations supported participants’ understanding of each mechanism and facilitated more reliable self-selection during the experimental procedure.

### Collection

In this study, the collection mechanism refers to the accumulation of virtual items or badges over time. Collecting has been identified as a motivational strategy that can support engagement in health-related behaviors, such as maintaining physical activity [28]. Our results show that participants scoring higher on conscientiousness were 1.87 times more likely to select the collection mechanism than those with lower levels of this trait.

These findings are largely consistent with the existing literature, which reports a general preference for collection-based mechanisms across most Big Five personality traits in the preference matrix M1 [9,24,29-31]. Notably, however, none of the previously reviewed studies identified a specific preference for collection among highly conscientious individuals. As such, the present result constitutes a novel contribution to the preference matrix by establishing a new, trait-specific association between conscientiousness and the collection mechanism.

### Competition

In this study, the competition mechanism is defined as enabling users to engage in competitive interaction with others in order to encourage the target behavior [23].

According to the preference matrix M1, competition is associated with all 5 Big Five personality traits. However, our results reveal a more differentiated pattern. Individuals scoring high in conscientiousness (OR 3.22, 95% CI 1.73-6.00) and agreeableness (OR 1.93, 95% CI 1.08-3.45) were significantly more likely to select competition as a motivating mechanism for maintaining their health compared with participants scoring lower on these traits. In contrast, participants with higher levels of extraversion (OR 0.43, 95% CI 0.25-0.74) and neuroticism (OR 0.52, 95% CI 0.30-0.89) were significantly less likely to select this mechanism.

Consistent with these findings, the ordinal regression analysis showed that participants with higher conscientiousness scores rated the competition mechanism as more motivating (OR 1.63, 95% CI 0.08-0.89), whereas those with higher neuroticism scores assigned lower motivation ratings (OR 0.71, 95% CI 0.70-0.01).

These divergent results suggest a controversial or polarized preference for competitive mechanisms. Qualitative feedback indicated that some participants favored individual-based mechanisms that emphasize personal effort rather than social comparison or rivalry. This interpretation aligns with previous research, which reports similar reservations toward competitive features in health-related apps, particularly when users prefer self-directed rather than socially driven motivation [10].

*No comparison with others because it stresses me out if I don't win. I'm more motivated by the idea of doing myself good.*

[P14]

*I choose for myself, not for others, so there’s no competition or comparison with others.*

[P39]

*I've excluded those that involve other people because I prefer autonomy.*

[P129]

*I no longer have anything to prove to anyone but myself.*

[P134]

*The individuality aspect. In my opinion, when you do something, you do it for yourself and not for others.*

[P112]

*I think the principle of fitness should be personal and not a competition with others.*

[P140]

While other participants said they found competition more motivating:

*I find competition very enjoyable, it pushes me to excel.*

[P44]

*Comparing myself to others pushes me to do better.*

[P176]

*I'm very competitive and I like it when people show me that I've made progress. It encourages me to keep going.*

[P192]

The preference matrix (M1) further indicates that individuals scoring high in agreeableness and conscientiousness tend to prefer competition [8,10,32]. However, it also reports a preference relation with extraversion [9,10,24] and neuroticism [33], whereas our results show that individuals high in these 2 traits selected the competition mechanism less frequently. This discrepancy is noteworthy, as the preference matrix (M1) is based on evidence from three studies reporting a positive association between extraversion and competition, which contrasts with the nonpreference pattern observed in our data. In addition, several studies examining competitiveness more broadly have documented a general attraction to competitive contexts among extraverted individuals, further emphasizing the unexpected nature of our findings [34,35]. One possible explanation lies in the heterogeneity of the extraversion construct itself: preferences for competitive environments appear to vary depending on specific subfacets of extraversion [36]. Individuals scoring higher on friendliness (“make friends easily” and “often feel uncomfortable around others”) or cheerfulness (“have a lot of fun” and “am not easily amused”) tend to show reduced interest in competitive dynamics. In contrast, those characterized by higher assertiveness (“take control of things” and “wait for others to lead the way”) or excitement-seeking (“love action” and “dislike loud music”) display stronger preferences for competition and achievement-oriented contexts [36]. These nuances suggest that the link between extraversion and preference for competitive mechanisms is not uniform and may be facet-dependent, an aspect that warrants deeper investigation in future research.

By contrast, the preference matrix (M1) also reports a nonpreference relation for individuals with higher levels of neuroticism profiles [32], a pattern that is corroborated by the results of our experiment.

### Rewards

The reward mechanism involves the provision of virtual incentives to users for completing a target behavior [23]. It differs from the collection mechanism in that rewards are not necessarily accumulated as part of a predefined or structured set of items.

In our study, individuals with higher levels of neuroticism (OR 1.97, 95% CI 1.36-2.86) and conscientiousness (OR 2.36, 95% CI 1.53-3.63) were significantly more likely to select the reward mechanism. In addition, these participants assigned higher motivation scores to this mechanism (OR_Conscientiousness_1.66, 95% CI 0.16-0.85; OR_Neuroticism_1.38, 95% CI 0.03-0.61).

These findings are consistent with the preference matrix (M1), which reports established preference relations between the reward mechanisms and both conscientiousness and neuroticism across multiple studies [24,30,32,33,37,38].

### Big Five Dimensions

Notably, conscientiousness emerged as a significant predictor across all 3 mechanisms identified in this study. In contrast, no significant associations were observed for openness, despite prior literature suggesting preferences for certain mechanisms, such as collection and competition, among individuals high in this trait.

Similarly, no significant preference results were identified for extraversion, even though the preference matrix derived from the literature (M1) reports associations between extraversion and multiple mechanisms. A comprehensive summary of the experimental findings alongside the preference matrix is presented in Table 10.

**Table 10.** Preferred and nonpreferred mechanisms for each Big Five dimension.

Big Five dimension	Mechanisms preferred on this study	Mechanisms nonpreferred on this study	Mechanisms preferred on preference matrix (M1)	Mechanisms nonpreferred on preference matrix (M1)
Openness to experience	—^a^	—	Self-monitoring, punishment, social comparison, social support, competition, cooperation, collection, rewards, and customization	Rewards, competition, and cooperation
Agreeableness	*Competition* ^b^ ^,^ ^c^	—	Prompts and cues, demonstration of the behavior, self-monitoring, punishment, social comparison, social support, progression, competition^b^, cooperation, collection, rewards, challenge, and customization	Social comparison and *competition*
Conscientiousness	Collection, competition^b^, and rewards^b^	—	Demonstration of the behavior, self-monitoring, progression, competition^b^, rewards^b^, quest, and challenge	Self-monitoring, cooperation, and customization
Extraversion	—	*Competition*	Prompts and cues, demonstration of the behavior, self-monitoring, punishment, social comparison, social support, progression, *competition*, cooperation, collection, rewards, quest, challenge, avatar, and customization	Customization
Neuroticism	Rewards^b^	*Competition* ^b^	Self-monitoring, social comparison, social support, progression, *competition*, cooperation, collection, rewards^b^, and customization	Competition^b^

a Not applicable.

b Indicates findings consistent with the literature

c *Italicized text* indicates findings inconsistent with the literature.

### Applying the Preference Matrix in Practice

Implementing the preference matrix involves 2 main steps: identifying the user profile and tailoring the app content accordingly. User profiling traditionally relies on validated personality questionnaires (eg, BFI-10 and NEO-PI-R); however, these instruments can be time-consuming and may discourage user participation. As an alternative, automated profiling approaches based on smartphone usage patterns [39,40] or social media data [41-43] have shown promising results, although privacy concerns remain a major limitation. Such approaches require access to personal data, sometimes including sensitive inputs such as audio or video, which many users are reluctant to share [44]. Moreover, extensive data collection may increase perceptions of intrusiveness or manipulation, potentially undermining user trust and the perceived validity of behavior-based personalization [44]. To address these concerns, we recommend making personalization optional, offering users the choice to complete a brief, validated personality questionnaire if they opt in, and implementing strict ethical safeguards to ensure responsible data use. In particular, personality-related data should be collected solely for the purpose of enabling individualized personalization within the app, should not be repurposed for any secondary uses or shared with third parties, and should be handled with full transparency. Users must be clearly informed about what information is being collected, how it will be processed, and for what specific purpose (to tailor the app’s features to their personality profile), thereby promoting informed consent, user autonomy, and trust.

Once the profile is established, app content can be adapted by selecting mechanisms aligned with the user’s dominant personality traits, as defined by the preference matrix. For example, a user scoring high in conscientiousness might be shown mechanisms emphasizing rewards, competition, and collection, while competitive features could be intentionally avoided for a more extraverted user. This automated adaptation enhances user engagement by aligning motivational strategies with intrinsic preferences derived from psychological profiling.

However, to ensure that personalization remains user-centered and flexible, the system should also allow users to manually adjust or refine these automatically suggested mechanisms. After reviewing the proposed configuration, users can confirm, modify, or reject specific mechanisms based on their subjective preferences or contextual needs. This hybrid approach, combining data-driven profiling with user autonomy, maximizes both the accuracy and acceptability of personalization. It also mitigates potential mismatches between predicted and actual motivation, ensuring that the final app configuration genuinely reflects the user’s individual preferences.

### Limitations

The requirement for participants to select exactly 5 mechanisms out of 15 constitutes a methodological limitation. Some participants may have selected mechanisms they did not find particularly motivating to reach the required number, while others may have wished to select more than 5 mechanisms but were constrained by the study design. This forced-choice approach may, therefore, have influenced the distribution of selected mechanisms and attenuated individual preference differences.

In addition, our study sample was not fully representative of the general population, as it included a high proportion of university students, who are typically younger (mean_age_ 29.42, SD_age_ 10.41). Such demographic homogeneity may restrict the extent to which these findings can be generalized to other age groups or populations. Nevertheless, prior research on digital behavior changes and web-based health interventions suggests that age does not substantially moderate the effects of these interventions on health-related behaviors [45,46]. Systematic reviews have similarly reported that demographic variables such as age, sex, and education tend to have weak or inconsistent moderating influences on the effectiveness of these techniques [45]. Moreover, individuals most inclined to engage with health apps are generally younger and possess higher levels of education and income [47], which aligns closely with the characteristics of our sample and supports the external validity of our findings. Future research should, however, aim to include older adults and individuals less familiar with digital technologies to examine whether similar patterns of preference and engagement are observed in these groups. This population is particularly relevant to investigate, as older adults also represent an active and growing user base of mHealth apps, often using them for health monitoring, disease prevention, or physical activity tracking. Recent evidence from a comprehensive scoping review underscores the growing importance of this demographic and highlights that addressing key barriers, such as evolving capability requirements, cost, privacy concerns, and age-related design assumptions, can substantially enhance meaningful engagement. It also emphasized the importance of empowering older adults through learner-centered, need-based digital education [48]. Building on these insights, future studies should explore how mHealth apps can be designed to support sustained digital engagement among older adults and to better capture the diversity of their experiences and values in technology use.

Another limitation concerns the personality assessment instrument used in this study. We used the BFI-10-Fr, an ultra-short version of the Big Five Inventory, to minimize participant burden given the overall length of the online questionnaire. Although the BFI-10-Fr provides a practical and time-efficient measure, previous research has noted that its internal reliability can be relatively low due to the limited number of items per trait [49]. Nevertheless, according to the original validation study by Rammstedt and John [49], the BFI-10 demonstrates satisfactory test–retest stability and acceptable construct validity, supporting its suitability for large-scale or time-constrained surveys. Future studies should, however, consider replicating these analyses using a more comprehensive personality inventory, such as the Big Five Inventory-44 or NEO-PI-R, to ensure higher internal consistency and further validate the robustness of the associations observed.

We also observe that, despite our sample size calculation, it appears that it did not allow for proper discrimination, as we obtained significant results for only 3 out of 14 mechanisms. Therefore, this calculation should be reconsidered.

It was not feasible to assess the customization mechanism, given its expansive scope and the consequent challenges in representing it in the format of a conventional mockup screen.

Although the regression analyses yielded statistically significant results for several mechanisms, the explanatory power of the models remained relatively modest. For instance, the proportion of variance explained ranged from 6% to 10% for the collection mechanism, and from 5% to 9% for the ordinal regressions predicting motivation scores. Even for the most predictive models, such as competition (15%‐33%) and Rewards (16%‐27%), a substantial portion of the variance remained unexplained. This indicates that while personality traits, particularly conscientiousness and neuroticism, appear to play a meaningful role in the selection of gamification mechanisms, other unmeasured variables likely contribute to these motivational choices. Therefore, the practical significance of these findings should be interpreted with caution: the Big Five dimensions alone may not fully account for users’ preferences, which are likely shaped by situational, contextual, or motivational factors beyond personality. Future studies could aim to include such variables, or use alternative modeling approaches (eg, interaction terms or structural models) to capture more of the underlying variance in mechanism selection.

### Conclusions

The entire preference matrix could not be validated with experimental data. Significant relations were identified for three mechanisms only: collection, competition, and rewards. Most of these preference relations are consistent with those reported in the literature-based preference matrix (M1), apart from the preference relation for the collection mechanism and conscientiousness and the nonpreference relation for competition regarding the extraversion trait, which is significant in this study but, according to the preference matrix M1, is a preference relation. The neuroticism trait is significant for competition as nonpreferred, yet the preference matrix studies demonstrated both a preference and a nonpreference relation for this trait. Considering these findings, it can be concluded that a nonpreference relation is corroborated. It would be interesting to replicate this study with a larger, less student-dominated panel to achieve better representation and more significant results on mechanism preference relations by Big Five traits.

## Supplementary material

10.2196/78939 Multimedia Appendix 1 Relation between Big Five traits and mechanisms.

10.2196/78939 Multimedia Appendix 2 Print version of the online questionnaire.

10.2196/78939 Checklist 1 CHERRIES checklist.

## Abbreviations

BFI-10: Big Five Inventory-10
BFI-10-Fr: Big Five Inventory-10, French version
CHERRIES: Checklist for Reporting Results of Internet E-Surveys
mHealth: mobile health
NEO-PI-R: Revised NEO Personality Inventory
OR: odds ratio
