# Supplementary material for: Personalizing Mobile Apps for Health Behavioral Change According to Personality: Cross-Sectional Validation of a Preference Matrix
Source: JMIR Hum Factors. 2026 Apr 22;13:e78939. doi: 10.2196/78939 (PMC13102323; doi:10.2196/78939)
Supplement: Multimedia Appendix 1 [file humanfactors-v13-e78939-s001.docx]

**Table 1. Relation between big five traits and mechanisms**

| Personality profile Big Five | BCT^a^ mechanisms | | | | | | Game elements | | | | | | | | App mechanism |
| --- | --- | --- | --- | --- | --- | --- | --- | --- | --- | --- | --- | --- | --- | --- | --- |
|  | Prompts and cues | Demonstration of the behavior | Self-monitoring | Punishment | Social comparison | Social support | Progression | Competition | Cooperation | Collection | Rewards | Quest | Challenge | Avatar | Customization |
| Openness to experience |  |  | ++^b^ | + | + | ++ |  | +++ -^c^ | + - | ++ | ++ - |  |  |  | ++  + |
| Agreeableness | + | + | +++ | ++ | ++ - | +++ | ++ | ++ - - | +++ | ++ | ++++ |  | ++ |  | +++ |
| Conscientiousness |  | + | + - |  |  |  | + | ++ | - - |  | ++ | + | + |  | - |
| Extraversion | + | + | ++ | + | +++ | ++ | ++++ | +++ | ++ | + | +++++ | + | ++ | + | +++  - |
| Neuroticism |  |  | + |  | + | + | + | + - | + | ++ | ++++ |  |  |  | + |

^a^BCT: behavior change techniques.

^b^+: 1 study with a preference relation.

^c^–: 1 study with non-preference relation.
