# Supplementary material for: Personalizing Mobile Apps for Health Behavioral Change According to Personality: Cross-Sectional Validation of a Preference Matrix
Source: JMIR Hum Factors. 2026 Apr 22;13:e78939. doi: 10.2196/78939 (PMC13102323; doi:10.2196/78939)
Supplement: Multimedia Appendix 2 [file humanfactors-v13-e78939-s002.docx]

**Multimedia Appendix 2**

Print version of the online questionnaire

Consent form

**Project title**: Evaluation of a mobile application personalization model for behavior change in the health domain
**Research objective**: This research aims to evaluate if our model, based on the literature, correctly links the user profiles and the different functionalities identified. Indeed, we want to verify that people prefer a certain type of functionality according to their profile. **Procedure:** You will have to fill in this online questionnaire. After accepting this form, you will have to answer some questions. First, you will have to answer demographic questions. Then, you will have to tell us which functionality for a mobile application to get you back in shape you prefer among the features presented. The questionnaire is entirely online and will take about 15 minutes to complete.
**Disadvantages and possible risks for participants**: There are no drawbacks or risks.
**Data protection:** The data collected (your answers to the questions) will be anonymous, meaning we will have no way to identify you. The data will be stored on a USB key in a locked drawer in our offices and on a secure server. Anonymized data may be reused in future research and/or shared with other researchers. This consent form will be kept for 5 years in a locked drawer in Mr. Gilles Falquet's office. **Access to research results for participants**: If you are interested in the research results, you can contact Mrs. Laëtitia Gosetto from 01.12.2022. Due to the anonymization of the data, no individual results can be transmitted.
**Name and contact details:** In case of questions, you can contact: Gilles Falquet, Associate Professor, gilles.falquet@unige.chFrédéric Ehrler, Senior Lecturer frederic.ehrler@hcuge.chGosetto Laëtitia, Assistant, laetitia.gosetto@unige.ch

Based on the above information, I confirm my agreement to participate in the research "Evaluation of a personalization model of a mobile application for behavioral change in the field of health":
I authorize the use of the data for scientific purposes and the publication of the results of the research in scientific journals or books, with the understanding that the data will remain anonymous and that no information will be given about my identity;

- Yes (1)
- No (2)

I authorize the use of the data for educational purposes (courses and training seminars for students or professionals subject to professional secrecy).

- Yes (1)
- No (2)

I have voluntarily chosen to participate in this research. I have been informed that I may withdraw at any time without providing any justification. I agree with all of the above by continuing to the next page.

Demographic issues

How old are you?

________________________________________________________________

What is your gender?

- Male (1)
- Female (2)
- Other (3)
- Do not wish to comment (4)

What is your highest level of education or degree obtained?

- elementary school (1)
- cycle / college (in France) (2)
- CFC / BEP (in France) (3)
- Maturity / Baccalauréat (in France) (4)
- Bachelor / licence (in France) (5)
- Master (6)
- Doctorate (7)
- Other (8) ________________________________________________

How comfortable are you with using mobile apps on your smartphone?

- Very comfortable (1)
- at ease (2)
- a little comfortable (3)
- not at all comfortable (4)

Are you interested in eating healthier?

- Not at all (1)
- A little (2)
- Enough (3)
- A lot (4)

Overall, how would you rate your eating habits?

- Very Unhealthy (1)
- Unhealthy (5)
- Reasonable (2)
- healthy (3)
- Very healthy (4)

How often do you cook your meals?

- never (1)
- 1-2 times a week (2)
- Almost every day (3)
- Everyday (4)

Do you ever take care of food shopping?

- yes (1)
- No (2)

What is your average personal budget per month for food (including catering and food shopping)?

- Less than 100 CHF (1)
- 100 to 200 CHF (2)
- 200 to 400 CHF (3)
- 400 to 600 CHF (4)
- 600 to 800 CHF (5)
- more than 800 CHF (6)

Are you interested in getting more exercise daily?

- Not at all (1)
- A little (2)
- Enough (3)
- A lot (4)

Approximately how much time per week do you spend in physical activity (e.g., walking, biking, sports in general)?

- 0 (1)
- 30 minutes (2)
- 1h (3)
- 2h (4)
- 3h (5)
- More than 3 hours (6)

Use of mobile app for health

Have you used a mobile application for health? (to exercise, eat healthier, measure steps, etc.)

- yes (1)
- Perhaps (2)
- No (3)

If so, do you still use them now

- Yes (1)
- No (2)

Post this question:

If yes, are you still using them now = No

| 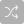 |
| --- |

What features did you use most often?

- Reminder (4)
- see its results/statistics (5)
- challenges (6)
- advice/recommendation (7)
- course/lesson (8)
- lens mount (10)
- motivational messages (11)
- return on its activities (12)
- History (13)
- awards (14)
- performance sharing (15)
- comparison between people (16)
- competition between people (17)
- other (9) ________________________________________________

Post this question:

If yes, are you still using them now = No

| 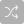 |
| --- |

What features did you never use?

- Reminder (4)
- see its results/statistics (5)
- challenges (6)
- advice/recommendation (7)
- course/lesson (8)
- lens mount (10)
- motivational messages (11)
- return on its activities (12)
- History (13)
- awards (14)
- performance sharing (15)
- comparison between people (16)
- competition between people (17)
- other (9) ________________________________________________

Post this question:

If yes, are you still using them now = Yes

| 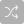 |
| --- |

What features do you use most often?

- Reminder (4)
- see its results/statistics (5)
- challenges (6)
- advice/recommendation (7)
- course/lesson (8)
- lens mount (10)
- motivational messages (11)
- return on its activities (12)
- History (13)
- awards (14)
- performance sharing (15)
- comparison between people (16)
- competition between people (17)
- other (9) ________________________________________________

Post this question:

If yes, are you still using them now = Yes

| 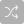 |
| --- |

What features do you ever use?

- Reminder (4)
- see its results/statistics (5)
- challenges (6)
- advice/recommendation (7)
- course/lesson (8)
- lens mount (10)
- motivational messages (11)
- return on its activities (12)
- History (13)
- awards (14)
- performance sharing (15)
- comparison between people (16)
- competition between people (17)
- other (9) ________________________________________________

Profile measurements

We will now ask you to answer some questions that will allow us to measure your profile.
Please select the most appropriate answer for you.

Big Five- BFI-10

You will find several qualifiers that may or may not apply to you.
**Select the most appropriate answer for each of these qualifiers.**

I see myself as someone who ...

|  | strongly disapproves (1) | disapproves a little (2) | neither approves nor disapproves (3) | approves a little (4) | strongly approves (5) | can't tell (0) |
| --- | --- | --- | --- | --- | --- | --- |
| is reserved (1) |  |  |  |  |  |  |
| generally trusts others (2) |  |  |  |  |  |  |
| works conscientiously (3) |  |  |  |  |  |  |
| is "relaxed", unwound, handles stress well (4) |  |  |  |  |  |  |
| has a great imagination (5) |  |  |  |  |  |  |
| is sociable, outgoing (6) |  |  |  |  |  |  |
| tends to be lazy (7) |  |  |  |  |  |  |
| is easily anxious (8) |  |  |  |  |  |  |
| has little interest in anything artistic (9) |  |  |  |  |  |  |

Perception of the social norm

Indicate your level of agreement with the following statements:

|  | Strongly disagree (1) | Disagree (2) | Somewhat disagree (3) | Neither agree nor disagree (4) | Somewhat agree (5) | Agree (6) | Strongly agree (7) |
| --- | --- | --- | --- | --- | --- | --- | --- |
| Most of the people who are important to me would approve of me getting in shape (1) |  |  |  |  |  |  |  |
| Most people like me get back in shape (2) |  |  |  |  |  |  |  |

Hexad Scale

Indicate your level of agreement with the following statements:

|  | Strongly disagree (15) | Disagree (16) | Somewhat Disagree (17) | Neither agree nor disagree (18) | Somewhat agree (19) | Agree (20) | Strongly agree (21) |
| --- | --- | --- | --- | --- | --- | --- | --- |
| I am happy if I can help others (1) |  |  |  |  |  |  |  |
| I like to help others find their way in new situations (2) |  |  |  |  |  |  |  |
| I like to share my knowledge (3) |  |  |  |  |  |  |  |
| The well-being of others is important to me (4) |  |  |  |  |  |  |  |
| Interaction with others is important to me (5) |  |  |  |  |  |  |  |
| I like being part of a team (6) |  |  |  |  |  |  |  |
| It is important for me to feel part of a community (7) |  |  |  |  |  |  |  |
| I like group activities (8) |  |  |  |  |  |  |  |
| It is important for me to follow my own path (9) |  |  |  |  |  |  |  |
| I often let my curiosity guide me (10) |  |  |  |  |  |  |  |
| I like to try new things (11) |  |  |  |  |  |  |  |
| It is important for me to be independent (12) |  |  |  |  |  |  |  |
| I like to overcome obstacles (16) |  |  |  |  |  |  |  |
| It is important for me to always complete my tasks completely (17) |  |  |  |  |  |  |  |
| It is difficult for me to let go of a problem before I have found a solution (18) |  |  |  |  |  |  |  |
| I like to master difficult tasks (19) |  |  |  |  |  |  |  |
| I like to provoke (20) |  |  |  |  |  |  |  |
| I like to challenge the status quo (21) |  |  |  |  |  |  |  |
| I consider myself a rebel (22) |  |  |  |  |  |  |  |
| I don't like to follow the rules (23) |  |  |  |  |  |  |  |
| I like competitions where you can win a prize (24) |  |  |  |  |  |  |  |
| Rewards are a great way to motivate me (25) |  |  |  |  |  |  |  |
| The return on investment is important to me (26) |  |  |  |  |  |  |  |
| If the reward is sufficient, I will make the effort (27) |  |  |  |  |  |  |  |

Choice of features

Finally, we will show you some **examples of features for a mobile application that aims to motivate you to get fit by eating healthier and exercising more.**

You need to **imagine that you are a mobile application user with** this goal, and you can **select the features you prefer that** would motivate you the most to get fit.
We have designed **examples of features** that are neutral; of course, you need to project yourself on what the feature offers rather than how it looks.

Please look carefully at each of the following feature images.
You will then be asked to tell us which of these features would motivate you the most to get in shape.


We remind you that these are neutral examples and not based on their aesthetic aspects to make your choice, but on the function they offer.

AvatarAllows **you to**
Choose a fictitious avatar representing you in the application.


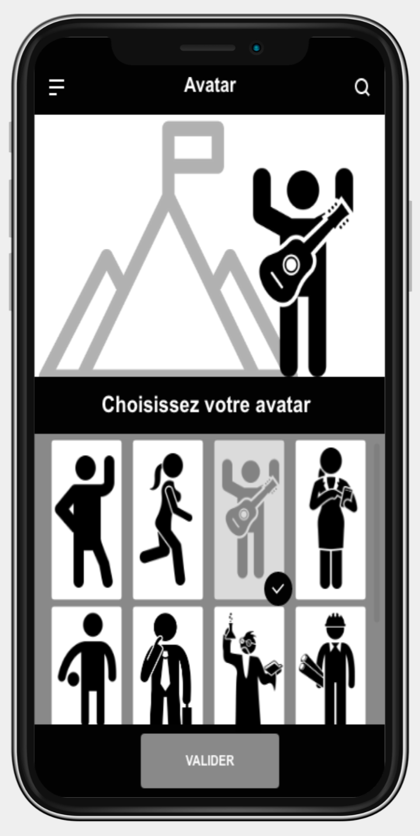


**Cooperation**
Allows you to work together to achieve a common goal


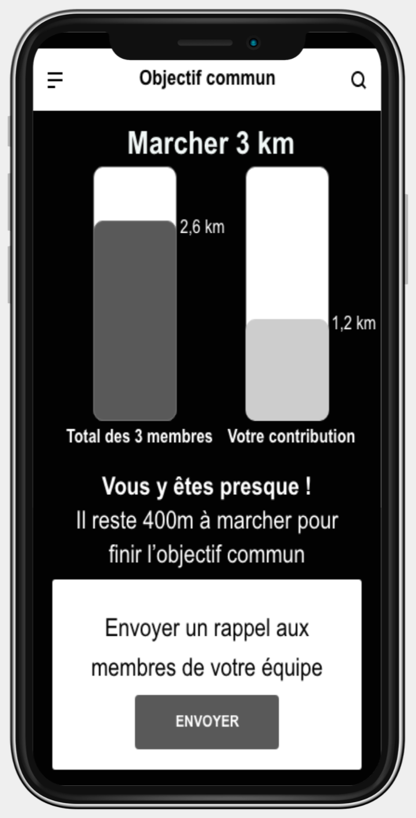


**Collectibles**
It allows you to collect virtual objects. In this example, it is a badge.


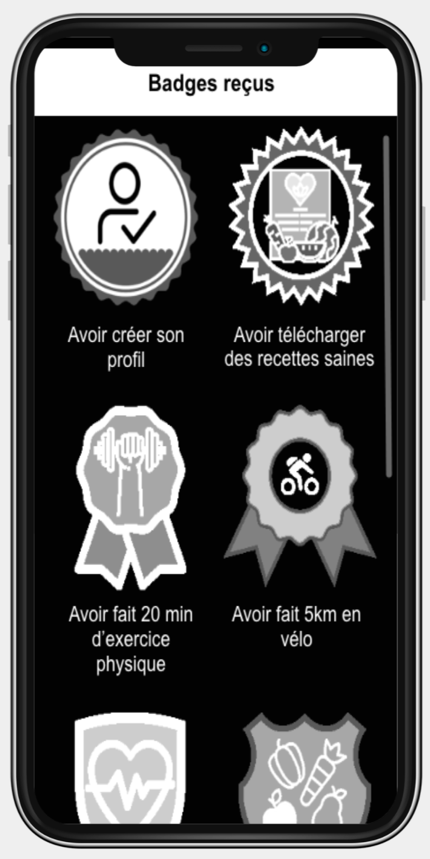


**Challenge**
It gives you a task whose difficulty depends on your skills, abilities, motivation, and knowledge.


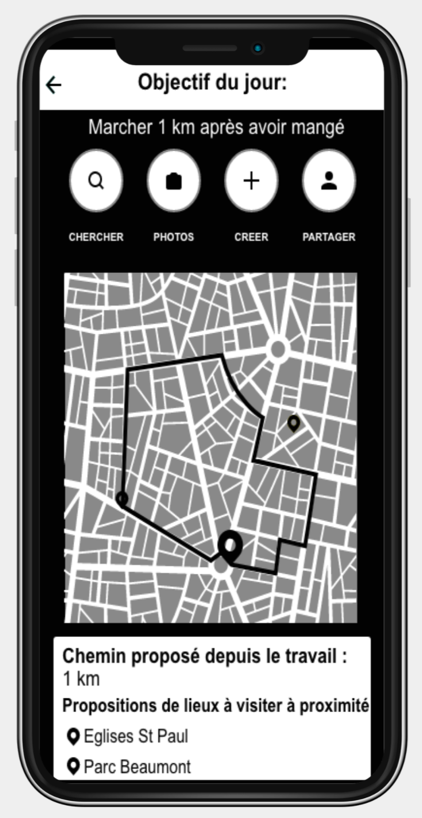


**Social comparison**
Allows you to compare your performance with other participants. This implies no losers or winners and no direct interaction with others.


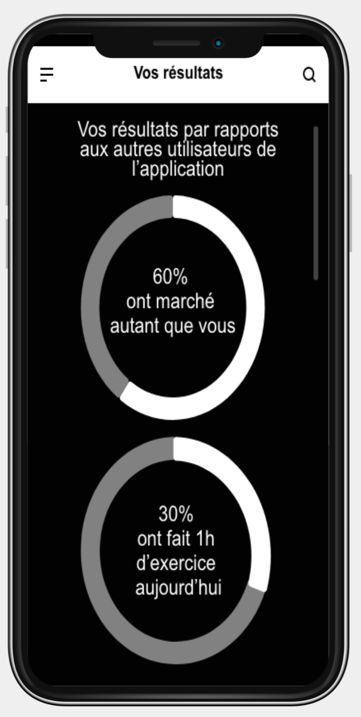


**Competition**
Allows you to compete with each other on your performance.


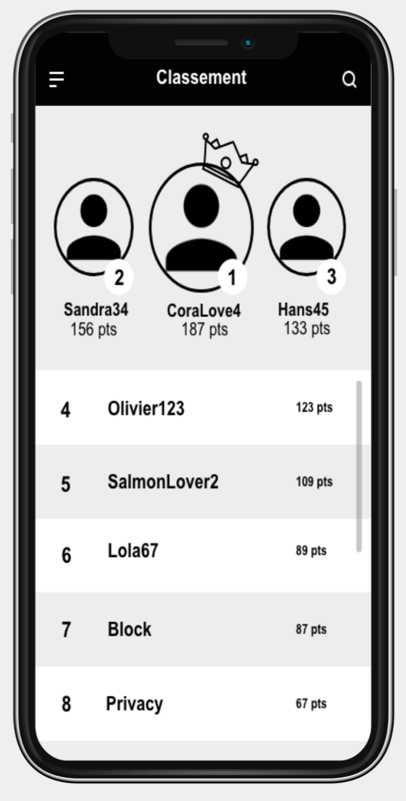


**Competitive Cooperation**
It allows you to work together to achieve a common goal while competing with others.


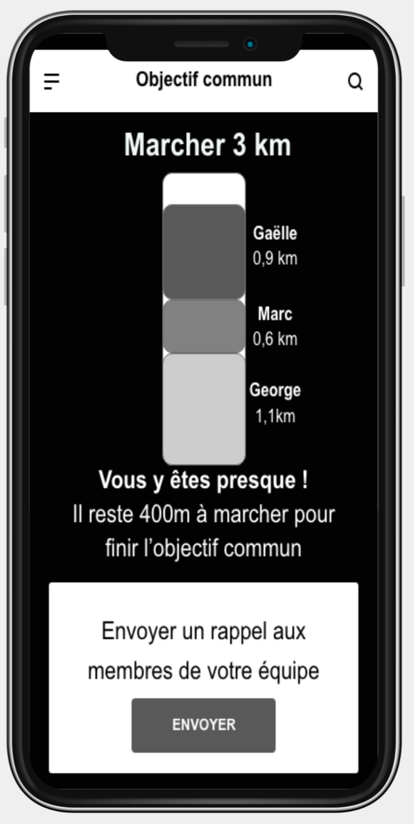


**Descriptive feedback**
You provide feedback on your behavior.


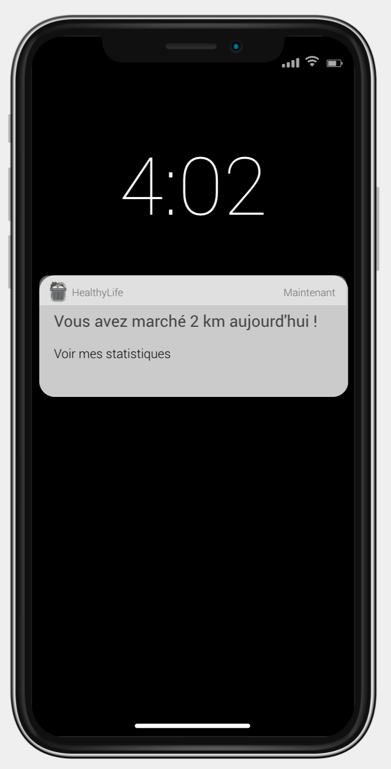


**Evaluative feedback**
Gives you feedback with an interpretation or judgment of your behavior.


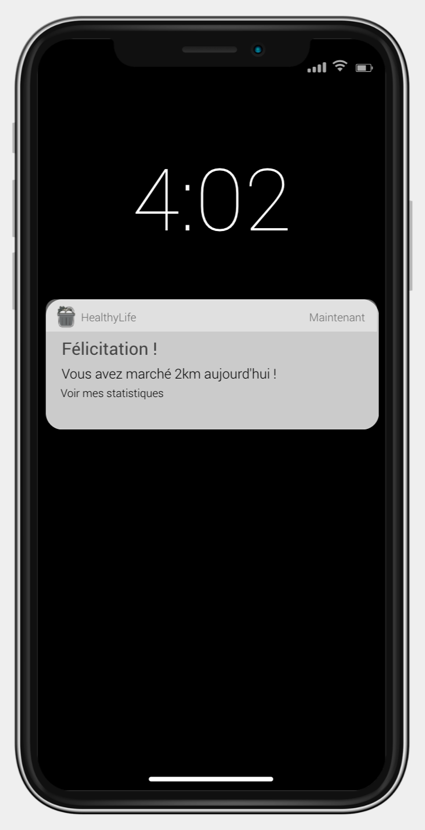


**Goal Setting**
Allows you to set your own goals.


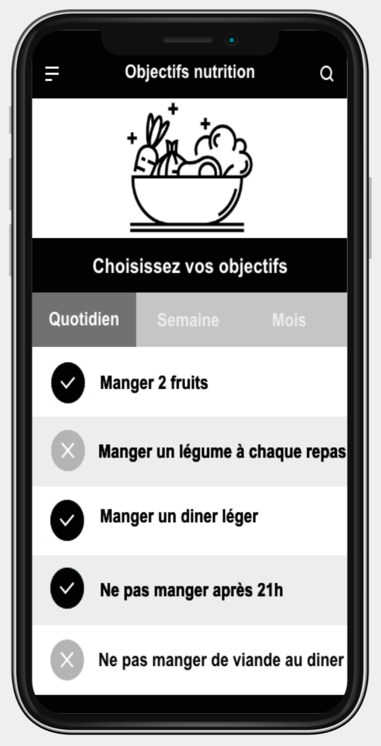


**Reminder**
Sends you reminders via notifications


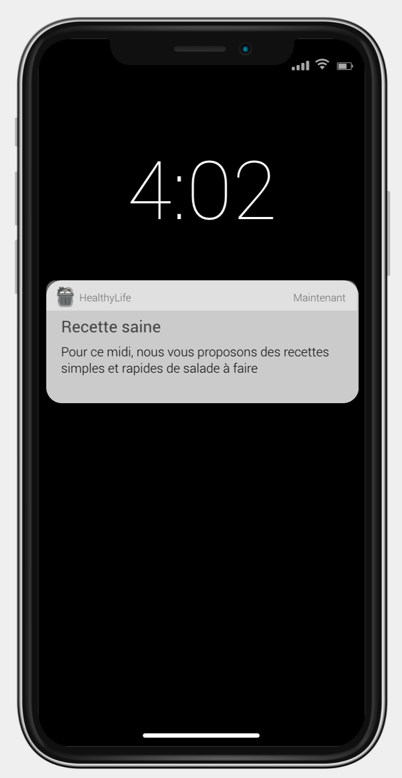


**Punishment**
Punishes you with virtual malus


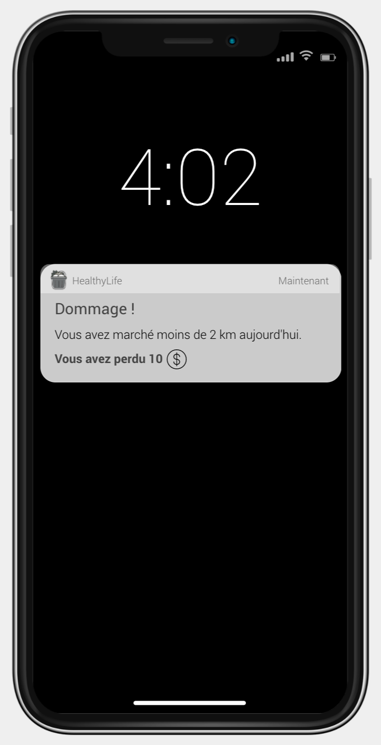


**Social NetworkIntegration of**
A mode that allows you to exchange with each other, post messages, images, etc.


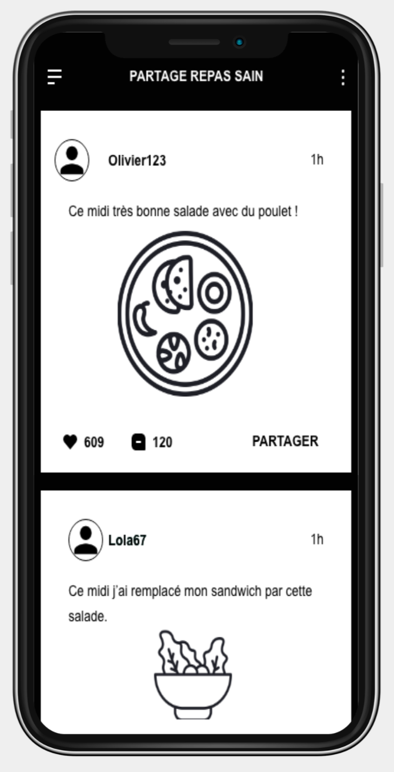


**Reward**
Gives you virtual or real rewards.


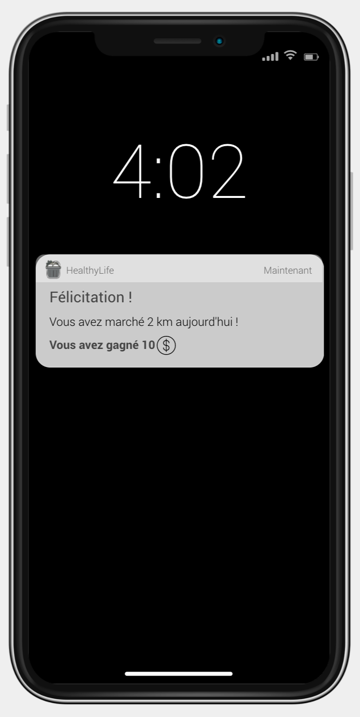


**Self-monitoring**
Allows you to observe your behavior; the system records and presents you in a readable way (with graphs, for example) your target behavior, for example, the number of steps taken.


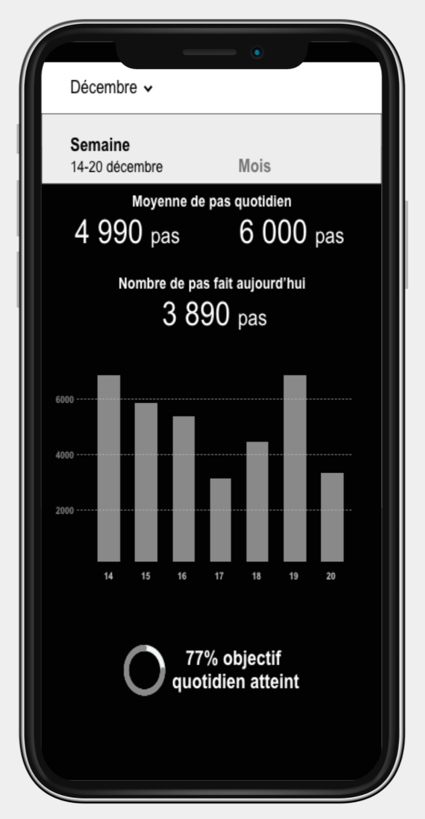


**Simulation**
A fictional representation of behavior. The goal is to see the consequences of a behavior.


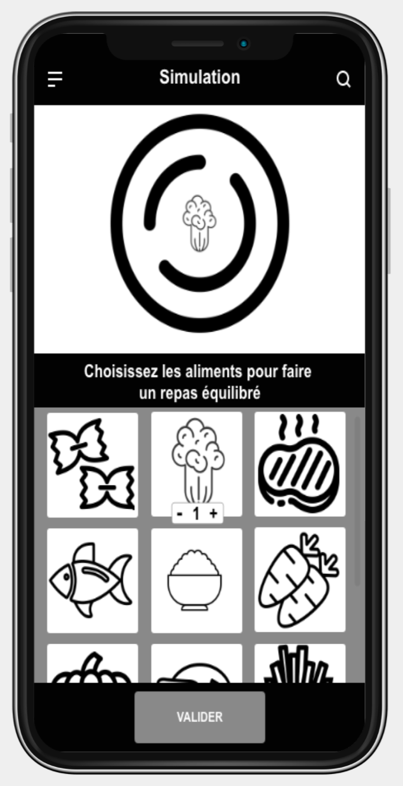


**Level and**
progressShows **you**
Your progress to the next level.


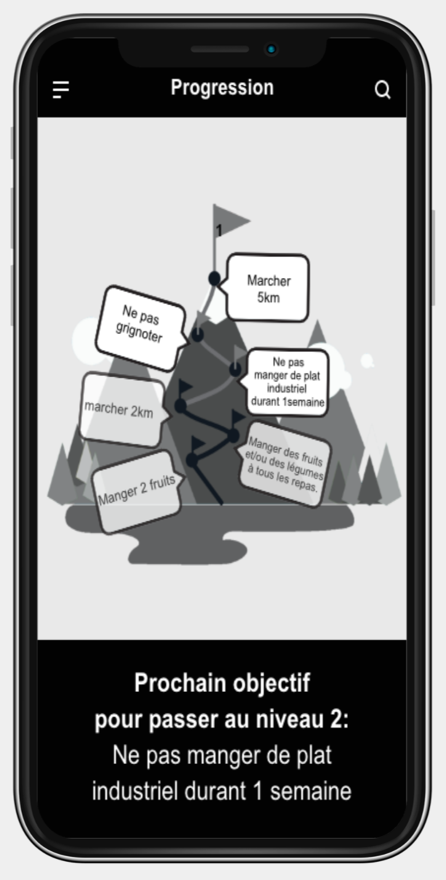


We'll ask you to tell us which of the features presented would motivate you the most to get in shape.

Then tell us on a score from 0 to 100 how much these features would motivate you, and briefly explain in writing the reason for this choice.

Choose **5** features among the ones presented to you that would motivate you the most to perform the behavior.
(be careful to choose only **5 features**)

- Avatar: choosing a virtual avatar (1)
- challenge: give the challenge to do (2)
- Collectibles: collect virtual objects (3)
- social comparison: compare performance between users without competition (4)
- competition: allows competition between users (5)
- Cooperation: working together to achieve a common goal (6)
- descriptive feedback: return with description (8)
- evaluative feedback: feedback with interpretation and/or judgment (9)
- goal setting: set your own goals (10)
- level and progress: shows you your progress to reach the next level (11)
- Reminder: send a reminder (12)
- punishment with virtual malus (13)
- social network: allows exchanging with other users (14)
- reward: gives virtual or real rewards (15)
- self-monitoring: shows you your performances collected by the app (16)
- simulation: shows you a simulation of a real behavior (17)
- Competitive cooperation: working together while competing with others (18)

Report Selected choices from "Choose 5 features from these features that you were presented with that would most motivate you to perform the behavior? (Be careful to choose only 5 features)."

On a score of 0 to 100, how much would this feature motivate you to get back into shape?

|  | 0 | 10 | 20 | 30 | 40 | 50 | 60 | 70 | 80 | 90 | 100 |
| --- | --- | --- | --- | --- | --- | --- | --- | --- | --- | --- | --- |

| Avatar : choice of a virtual avatar () | 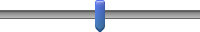 |
| --- | --- |
| challenge : give a challenge to do () | 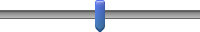 |
| Collectibles: collect virtual objects () | 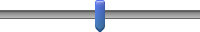 |
| social comparison: compare performance between users without competition () | 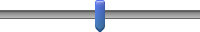 |
| competition: allows to compete between users () | 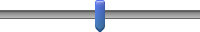 |
| Cooperation: working together to achieve a common goal () | 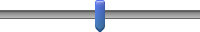 |
| descriptive feedback: return with description () | 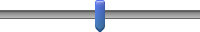 |
| evaluative feedback: feedback with interpretation and/or judgment () | 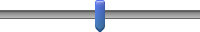 |
| goal setting: set your own goals () | 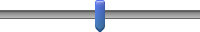 |
| level and progress : shows you your progress to reach the next level () | 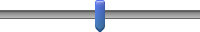 |
| Reminder: send reminder () | 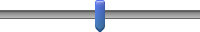 |
| punishment with virtual malus () | 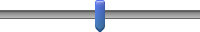 |
| social network : allows to exchange with other users () | 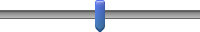 |
| reward : gives virtual or real rewards () | 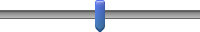 |
| self monitoring : shows you your performances collected by the app () | 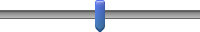 |
| simulation: shows you a simulation of a real behavior () | 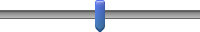 |
| Competitive cooperation: working together while competing with others () | 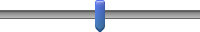 |

Is there a particular reason why you chose certain features?

________________________________________________________________

________________________________________________________________

________________________________________________________________

________________________________________________________________

________________________________________________________________

You can do so here if you have any remarks/comments to share with us.

________________________________________________________________
