# Supplementary material for: Personalizing Mobile Apps for Health Behavioral Change According to Personality: Cross-Sectional Validation of a Preference Matrix
Source: JMIR Hum Factors. 2026 Apr 22;13:e78939. doi: 10.2196/78939 (PMC13102323; doi:10.2196/78939)
Supplement: Checklist 1 [file humanfactors-v13-e78939-s003.pdf]

Checklist for Reporting Results of Internet E-Surveys (CHERRIES)

1. Design

Item	Explanation	Location in paper (pg)
**Describe survey design**	Describe target population and sample frame. Indicate whether the sample is a convenience sample (often the case in open surveys).	5

2. IRB Approval and Informed Consent Process

Item	Explanation	Location in paper (pg)
**IRB approval**	Mention whether the study has been approved by an Institutional Review Board (IRB).	5
**Informed consent**	Describe the informed consent process. Specify where participants were informed about survey duration, stored data, storage duration, investigator identity, and study purpose.	5
**Data protection**	If personal information was collected or stored, describe mechanisms used to protect against unauthorized access.	N/A

3. Development and Pre-testing

Item	Explanation	Location in paper (pg)
**Development and testing**	State how the survey was developed, including whether usability and technical functionality were tested before launching the questionnaire.	Protocol paper [25], Methods

4. Recruitment Process and Description of the Sample

Item	Explanation	Location in paper (pg)
**Open vs closed survey**	An open survey is accessible to any visitor; a closed survey is limited to a known sample (eg, password-protected).	5
**Contact mode**	Indicate whether the initial contact with potential participants was made online.	5
**Advertising the survey**	Explain how and where the survey was advertised (eg, newspapers, mailing lists, banner ads). The wording of the announcement should ideally be provided as an appendix.	5

5. Survey Administration

Item	Explanation	Location in paper (pg)
**Web/E-mail survey**	Specify whether the survey was hosted on a website or distributed via email. If email, indicate how responses were captured (manual entry or automated system).	5
**Context**	Describe the website or platform where the survey was posted, including its audience and potential influence on the sample.	5
**Mandatory/voluntary**	Indicate whether participation was mandatory or voluntary.	5
**Incentives**	Indicate whether incentives were offered (monetary or non-monetary).	5
**Time/date**	Specify the timeframe during which data were collected.	Protocol paper [25], Results
**Randomization of items**	State whether items or questionnaires were randomized to prevent bias.	Protocol paper [25], Methods
**Adaptive questioning**	Indicate whether conditional questions were used to reduce complexity.	Protocol paper [25], Appendix 2
**Number of items**	Specify the number of questionnaire items per page.	Protocol paper [25], Appendix 2
**Number of screens**	Indicate over how many pages the questionnaire was distributed.	Protocol paper [25], Appendix 2
**Completeness check**	State whether completeness or consistency checks were implemented before submission.	N/A
**Review step**	Indicate whether participants could review and change their answers before submission.	Protocol paper [25], Appendix 2

6. Response Rates

Item	Explanation	Location in paper (pg)
**Unique site visitor**	Define how unique visitors were identified (eg, IP addresses, cookies).	N/A
**View rate**	Ratio of unique survey visitors to unique site visitors.	N/A
**Participation rate**	Ratio of visitors who agreed to participate to visitors who saw the first survey page.	Protocol paper [25], Appendix 2
**Completion rate**	Ratio of participants who finished the survey to those who agreed to participate.	Protocol paper [25], Appendix 2

7. Preventing Multiple Entries

Item	Explanation	Location in paper (pg)
**Cookies used**	Indicate whether cookies were used to assign unique identifiers and prevent duplicate entries.	N/A
**IP check**	Indicate whether IP addresses were used to identify duplicate entries.	Protocol paper [25], Methods
**Log file analysis**	Describe whether log files were analyzed to identify duplicate responses.	N/A
**Registration**	For closed surveys, explain how login or registration prevented duplicate entries.	N/A

8. Analysis

Item	Explanation	Location in paper (pg)
**Handling incomplete questionnaires**	Indicate whether only complete questionnaires were analyzed or if partial responses were included.	Protocol paper [25], Methods
**Atypical timestamps**	Describe whether questionnaires submitted too quickly were excluded.	Protocol paper [25], Methods
**Statistical correction**	Indicate whether weighting or propensity score adjustments were used to correct for sample bias.	Protocol paper [25], Methods
